# Concentration, Source, and Total Health Risks of Cadmium in Multiple Media in Densely Populated Areas, China

**DOI:** 10.3390/ijerph16132269

**Published:** 2019-06-27

**Authors:** Kui Cai, Yanqiu Yu, Minjie Zhang, Kangjoo Kim

**Affiliations:** 1 Department of Geological Science & Engineering, Kunsan National University, Gunsan 573-701, Korea; 2 Institute of Geological Survey, Hebei GEO University, Shijiazhuang 050031, China; 3 Department of Environmental Engineering, Kunsan National University, Gunsan 573-701, Korea; 4 College of Resources, Hebei GEO University, Shijiazhuang 050031, China

**Keywords:** cadmium, SMLR, health, multiple media, cancer risk

## Abstract

Cadmium (Cd) is a non-essential and harmful element to humans. Cadmium contamination is a serious issue for human health, especially in densely populated agroecology areas. In this study, the investigation of an agroecology area was conducted to gain insight into the relationship between Cd in wheat and soil and then evaluate the Cd total risk for human health. The soil samples and their matching wheat samples, underground water samples, and atmospheric deposition (air) samples were collected from a wheat-growing area in an agroecology plain. The cadmium concentration in the four types of media, in order, was air > soil > wheat > water. The mean concentration of the geoaccumulation index (I_geo_) showed that the total Cd in soil (Cd-T) and Cdair reached a mild and moderate pollution level. The results of the correlation and principal component analysis (PCA) showed that the majority of Cdwheat originated from Cd-2 (exchangeable), Cd-4 (humic acid-bound), and Cd-7 (residual). Furthermore, the results of the stepwise multiple linear regression (SMLR) showed that three fractions were primarily controlled by Cd-T: clay, cation exchange capacity (CeC), and total organic carbon (TOC). In addition, the total cancer risk (CR) of Cd in multiple media was, in the order wheat > water > soil > air. It is noteworthy that the Cd content in underground water and wheat by the ingestion pathway posed cancer risks to the local residents and provided a comprehensive insight into multiple media environment management. Furthermore, it provides a very significant basic study for detailed research into the mobility and transformation for factions.

## 1. Introduction

Cadmium is a post-transition metal that has been found to exhibit biological toxicity that is “teratogenic, carcinogenic, and mutagenic.” It is easily absorbed by crops, causes harm to crop growth and development, affects crop quality and yield [1,2], and, as part of the food chain, leads to damage to the lungs, liver, kidneys, bones, and reproductive organs, causes toxic effects to the immune and cardiovascular systems, and is associated with various diseases [2,3,4,5]. The Agency for Toxic Substances and Disease Registry (ATSDR), Atlanta, GA, USA, lists Cd as the sixth most toxic substance that endangers human health. The United Nations Environment Program (UNEP) and the international committee on trace metals in occupational health also lists Cd as an environmental pollutant and a priority for study, while the World Health Organization (WHO) considers it a food pollutant [6].

At present, soil cadmium pollution has become a serious global environmental problem [7,8,9,10]. Cadmium pollution is found to varying degrees in the soils of Asia [2,9,11,12], North America [13,14], and Europe [10,15]. Cadmium appears in the soil after dissolution, condensation, chemical reaction, adsorption, complexation, and other reactions, thus affecting the migration and transformation of Cd in plants [16,17,18]. Studies have shown that for plants and animals, cadmium is a nonessential element. Compared with lead, copper, zinc, and arsenic, the amount of environmental Cd is much smaller, and is easily absorbed by plants and crops such as wheat and rice. It has a strong ability to become enriched, and easily enters the body through the food chain, resulting in a hazard to human health through water, air, and plants [19,20,21,22,23,24]. The symptoms of chronic Cd poisoning occur when contaminated agricultural products and drinking water are ingested. It has been found that the average daily Cd absorption is 25–75 μg/d [3], which is higher than the recommended tolerance of 10–35 μg/d recommended by the FAO/WHO [25,26]. Thus, people are already under a serious threat of environmental Cd pollution. As a key source of plant Cd in the soil environment, the study of the speciation of Cd is particularly critical.

The rate of increase of Cd in China’s grain production has become prominent in recent years. A communiqué [4] showed that sampling sites of Cd that exceeded the standard rate was 7%. Moreover, approximately 20 million hectares of arable land is contaminated with Cd, accounting for approximately one-fifth of the total cultivated soil. The amount of Cd entering farmland every year is as high as 1417 tons, among which Cd from atmospheric deposition is as high as 493 t [4,5,7]. The amount of Cd entering farmland due to sewage irrigation is 30 tons. Of the total 1417 tons of Cd entering farmland, 178 tons is taken away by various means each year; that is, only 13% of the Cd is exported per year, and 87% remains in the farmland [7,8,9]. With regard to the long-term coal-burning activities in the research area, the irrigation water used for agricultural production mainly comes from groundwater and untreated or lightly treated sewage, and the excessive use of chemical fertilizers has led to the enrichment of Cd ions in farmland, which has led to excessive Cd content in crops, endangering the health of residents in the irrigation area [8]. This has become one of the most crucial factors influencing the security of the rural ecological environment and restricting the sustainable development of agriculture.

At present, the research on pernicious metal Cd pollution in farmland soil has become a hot topic of research [19,20,24,27,28,29,30,31]. Most of the research areas have been concentrated on a small-scale range near the pollution source [11,12,18,22,23,28,31,32], and the mobility of the soil–wheat system and the total health risk of Cd in multiple media bases at the regional level has been rarely reported. On the other hand, cadmium regulatory limits have vast differences in agricultural soil worldwide due to the over protections concerning health risks [33]. Therefore, there is an urgent need to set up Cd regulatory limits scientifically. The objectives of this study were as follows: (1) grasp the concentration level of Cd in multiple media; (2) establish the relationship between soil speciation and wheat, and analyze the origin of Cd in wheat by using principal component analysis (PCA); (3) to further identify the effective physicochemical factors closely related to wheat by stepwise multiple linear regression (SMLR); and (4) evaluate the health risk level of Cd to adults and children in multiple media (wheat, soil, air, and groundwater). This research can not only offer a benefit reference for the setup fit of the local Cd standard limit, but the findings of this study will also offer significant support for the ecological security of multiple environments in Hebei Province, China.

## 2. Materials and Methods 

### 2.1. Study Area

**Figure 1:**
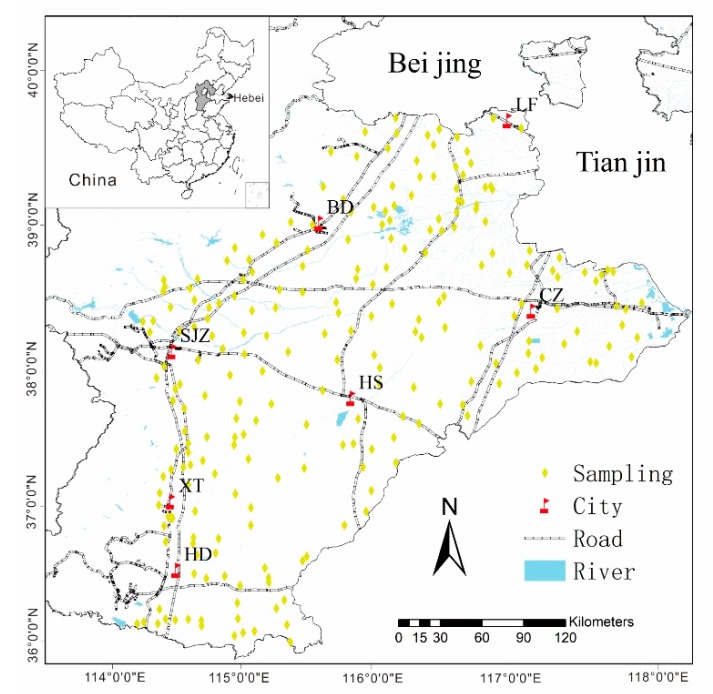
The sampling sites of wheat and corresponding soil in the study area. (BD: Baoding City, LF: Langfang City, CZ: Cangzhou City, SJZ: Shijiazhuang City, HS: Hengshui City, XT: Xingtai City, HD: Hangdan City). Water and wheat can lead to health risks in humans; air and soil do not pose health risks to humans. The arrows represent the relationship between each media.

The survey area lies in the mid-latitude region of China with geographic coordinates between 113°27’E~114°56’E and 36°02’N~39°03′N. The administrative city includes Shijiazhuang (SJZ), Cangzhou (CZ), Baoding (BD), Xingtai (XT), Langfang (LF), Handan (HD), and Hengshui (HS) (Figure 1), in the northeast direction around the Tianjin and Beijing areas. The total area is approximately 60,000 km^2^. This region is a semi-arid and semi-humid climate zone, with a warm temperate continental monsoon climate. It is rich in light and heat resources, with obvious seasonal changes and the same season of rain and heat. The winter is cold and dry, and the summer is hot and rainy. The annual average temperature is 11.5–12.5 °C, and annual precipitation is 500–800 mm, with the rainfall seasonal distribution mainly concentrated in the summer. The total population is approximately 55 million, and the crops consist mostly of summer corn and winter wheat, which is the most important farming system in this region. The sown area of wheat and corn accounts for approximately 80% of the total sown area of plain grain crops. The staple food of the local people is wheat (flour) [34]. 

### 2.2. Sampling 

A total of 230 surface cultivated soil samples were collected from Hebei Province, China (Figure 1). Corresponding wheat samples, 47 atmospheric deposition samples, and 234 underground water samples were collected in wheat-growing areas. These are described as follows.

1. Surface soil and wheat samples: soil and wheat seed were collected during the wheat ripening season. The weight of each soil sample was 1 kg, the sampling depth of a soil sample was 0–20 cm, and the weight of each fresh sample of crop grain was approximately 2 kg. The specimens were dried by air and sent to a laboratory for next step analysis.

2. Atmospheric deposition: the sampler was a polyethylene plastic bucket with a height of 28.5 cm and a circumference of 29.5 cm. After cleaning the barrel wall with distilled water, the sampler was placed on a rooftop approximately 5–10 m away from the ground and was fixed with a stainless-steel bracket away from obvious local pollution sources. Ethylene glycol (C_2_H_6_O_2_) was added to the polyethylene plastic bucket until 1 cm deep before it was placed at the sampling sites. The addition of ethylene glycol prevents freezing, keeps the bottom of the plastic bucket moist, and inhibits the growth of microorganisms and algae [35]. The settling period of receiving the atmospheric deposition was one year. When the atmospheric deposition was collected, we evaporated the suspension liquid from the bottom, and then cleaned the precipitation and plastic bucket wall with a wall brush. Finally, all of the suspension was transferred into a plastic bottle and packaged for testing and analysis.

3. The quality control was strictly carried out in the process of the groundwater sample collection. The site blank sample, site standard sample, and parallel sample were arranged in a 5% proportion, and repeat sampling was performed at abnormal test data sites. Hard glass bottles purchased uniformly were adopted, and samples were taken strictly according to the standard (HJ 493-2009) [36]. Freezers and car refrigerators were sent to the laboratory within the specified time.

### 2.3. Chemical Analysis and Quality Control 

#### 2.3.1. Soil and Air

The samples sent to the laboratory were screened with 20 mesh (<0.84), air-dried at room temperature, mixed, shrunk, and separated into soil samples of 200 g. The samples were crushed to 100 mesh (<0.25) with an agate pollution-free sample preparation machine and bagged for standby application.

The soil pH was determined in a suspension liquid of 2.5/1 (water/soil) by using a pH meter. total organic carbon (TOC) was determined by the potassium dichromate volumetric method. The analysis method of clay was the soil laser particle method; the cation exchange capacity (CeC) was measured by the ammonium acetate method. The detailed analysis method is referenced in the “Analysis Methods of Soil Agricultural Chemistry” [37].

A total of 0.5 g of the soil and air sample was digested with a mixture of HNO_3_, HClO_4_, and HF to measure the Cd. Cadmium was analyzed by atomic absorption spectrophotometry (M6MK2, Thermo Electron). The quality control method for sample analysis was strictly in accordance with [38]. The reference soils used to ensure the recovery within 95–110% included GBW 07402 (GSS-2) and GBW 07406 (GSS-6) (China National Center for Standard Materials). 

Seven sequential extraction processes were sequentially performed as follows: (1) water was used as an extractant to extract the water-soluble Cd; (2) exchangeable Cd was extracted with magnesium chloride (MgCl_2_) as an extractant; (3) carbonate-bound Cd was extracted with sodium acetate as an extractant; (4) humic acid-bound Cd was extracted with sodium pyrophosphate as an extractant; (5) Fe–Mn oxide-bound Cd was extracted with hydroxylamine hydrochloride as an extraction agent; (6) organic-bound Cd was extracted with hydrogen peroxide as an extractant; and (7) the residue Cd was extracted with hydrofluoric acid. Analyses were performed by graphite furnace atomic absorption spectrometry (GF-AAS).

The detection limits of each fraction of cadmium were as follows: water-soluble Cd 0.005 ug/g, exchangeable Cd 0.02 ug/g, carbonate-bound Cd 0.02 ug/g, humic acid-bound Cd 0.02 ug/g, Fe–Mn oxide-bound Cd 0.02 ug/g, organic matter-bound Cd 0.02 ug/g, and residual Cd 0.03 ug/g, respectively.

The accuracy of the Cd speciation analysis method is based on the total amount of elements in the soil as the standard, which is compared with the sum of all fractions to calculate the relative deviation RE% = [(CT − CS)/CT] × 100%, where the CT was the full content of Cd. CS was the sum of seven fractions, and the average relative deviation RE of Cd = 7.12% (RE ≤ 40%). The precision of the speciation analysis method was measured eight times with the same sample, and the (Relative Standard Deviation) RSD of each fraction repeat analysis was calculated with an RSD of <10%.

#### 2.3.2. Wheat

The grain samples were ground to 20–40 mesh (0.84–0.42 mm), and then the powdered (0.5 mg) samples were digested by 1V HClO_4_ and 3V HNO_3_ after mixture. Then, the concentration of Cd was determined by GF-AAS with a detection limit of 0.0001 mg/kg for Cd. The certified wheat material of GBW08503b (wheat flour) was the accuracy control [39].

#### 2.3.3. Underground Water

The analysis method of groundwater Cd samples was strictly in accordance with the GB/T 5750.6-2006 standard [40]. A certain amount of the water sample was taken for direct determination by using inductively coupled plasma mass spectrometry (Agilent 7500 Series ICP-MS, Agilent Technologies Company, Santa Clara, CA, USA). When the water sample was turbid, a 0.45-um membrane was used for filtration. The accuracy of the analytical method was tested by a standard addition recovery test (the standard addition amount was ten times the detection limit of the analytical method). The standard addition recovery rate was 90–110%. The precision of the analytical method was assessed by the repeated analysis of multiple samples. When the content of the tested components in the sample was higher (or equal) than ten times the detection limit, the relative standard deviation of the 12 tests was less than 15%.

### 2.4. Analysis of the Geoaccumulation Index

The assessment method of Cd in the cultivated soil and air of the study region were conducted using the geoaccumulation index (I_geo_) [41]. This approach not only reflects the natural distribution of trace metals in the soil, but also emphasizes the historical accumulation of pollution. The I_geo_ is also known as the Müller index, and takes full account of the influence of natural geology and human activities on trace metal pollution. It is widely applied to the evaluation of Cd accumulation in sediments and other substances. The equation is as follows:

(1) $$I_{geo}=log_{2}\frac{C_{Cd}}{1.5\times B_{Cd}}$$

where *C_Cd_* is the measured content of cadmium; 1.5 is the revised index; and *B_Cd_* is the local background value [42]. The classification standard is as follows: *I_geo_* = 0, no pollution; 0–1, mild to moderate; 1–2, moderate; 2–3, moderate–strong; 3–4, strong; 4–5, strong–extremely serious.

### 2.5. Bioconcentration Factor

The bioconcentration factor (BF) of trace metals refers to the ratio of the element content in a certain part of a plant to the corresponding element content in the soil. It reflects a certain degree of element migration in the soil–plant system [43,44]:

BF = Cdg/Cds(2)

where Cdg is the content of cadmium in wheat grain and Cds is the total content of trace metal cadmium in soil.

### 2.6. Input Fluxes of Atmospheric Deposition

For the cadmium content of the deposition in the sampling bucket and the area of the sampling bucket mouth, which we obtained by calculating the weight of Cd in the atmospheric deposition into the unit area, the calculation formula is

(3) $$F=\frac{C\times m}{{\left(\frac{P}{2\pi }\right)}^{2}\times \pi \times 100}$$

where F is the deposition fluxes of cadmium within one year (g/hm^2^ ∙ a); *C* is the content of cadmium in deposition (mg/kg); *m* is the total mass at a point within one year (g/a); *P* is the perimeter of the plastic bucket (29.5 cm); and *π* is the mathematical constant (3.14…).

### 2.7. Health Risk 

#### 2.7.1. Exposure Calculation

Health risk assessment takes the risk degree as the evaluating indicator, links the degree of different environmental contamination with human health, and quantitatively describes the health hazards of environmental pollutants on the human body. According to the United States Environmental Protection Agency (US EPA) risk assessment guidelines and health risk assessment model [25,26], the average daily exposure of adults and children was calculated by the three exposure pathways (oral, inhaled, and dermal) to Cd of wheat, soil, atmospheric dust, and groundwater in the studied area. The related calculation formula and parameter values are presented in Table 1.

#### 2.7.2. Health Risk Representation

Cadmium is a carcinogenic risk substance listed by the ATSDR. The potential non-carcinogenic risk was characterized by the health quotient (HQ) and health risk index (HI), and the calculation formula is shown below and in Table 1.

HQ_x_ = ADD_x_/RfD_x_ (4)

HI = HQ_ingestion_ + HQ_inhale_ + HQ_dermal_ (5)

where HQ_x_ (HQ_ingestion_, HQ_inhale_ and HQ_dermal_)is the Cd’s non-carcinogenic risk index under the x-exposure pathway; ADD_x_ is the exposure amount of the x-exposure(ADD_ingestion_, ADD_inhale_, and ADD_dermal_) pathway of element Cd, mg·kg^−1^·d^−1^; RfD_x_ (RfD_ingestion_, RfD_inhale_, and RfD_dermal_) is the reference dose of non-carcinogenic element Cd by the x-exposure pathways; and HI is the total non-carcinogenic risk index of Cd through three exposure pathways. When HQ_x_ or HI < 1, it indicates that the non-carcinogenic health risk is an acceptable risk level. HQ_x_ or HI > 1 indicates a non-carcinogenic health risk, and the greater the value, the greater the health risk [45,46,47]. The potential carcinogenic risk was characterized by risk index (CR), and the calculation formula is shown in Table 1.

CR_x_ = ADD_x_ × SF_x_ (6)

TCR = CR_ingestion_ + CR_inhale_ + CR_dermal_ (7)

where CR_x_ is the single cancer-causing risk index of carcinogenic elemental cadmium under the x-exposure pathway (CR_ingestion_, CR_inhale_, and CR_dermal_); SF_x_ is the slope factor of carcinogenic elemental Cd in the x-exposure pathways (SF_ingestion_, SF_inhale_), kg·d^−1^·mg^−1^; and TCR is the total carcinogenic risk index of elemental Cd through three exposure pathways. When CR < 10^−6^, it represents no cancer risk; 10^−6^ < CR < 10^−4^, human body tolerable cancer risk; CR > 10^−4^, human body cannot tolerate the risk of cancer [45,46,47]. The values and significance of the parameters are shown in Table S1.

### 2.8. Data Analysis

All of the statistical data for air (atmospheric deposition), wheat, soil, and water in this study were carried out using SPSS.24 (IBM, Armonk, NY, USA) including treated boxplot, the Pearson correlation analysis, and PCA maximum rotation variance method. The spatial distribution maps were made by ArcGIS 10.5 (ESRI, Redlands, CA, USA) including (Cd-1, water-soluble), (Cd-2, exchangeable), (Cd-3, carbonate-bound), (Cd-4, humic acid-bound), (Cd-5, Fe-Mn oxide-bound), (Cd-6, organic matter-bound), (Cd-7, residual), Cdwheat, Cdwater, Cdair, Cd-T and BF.

## 3. Results and Discussion

### 3.1. Cadmium Content and Its Speciation in Soil

The pH value of farmland soil in this area was 8.4, the soil organic carbon content was 1.04%, the CeC content was 11.08 mg/kg, and the clay content was 98.1 g/kg. The total and available content of cadmium in farmland soil is shown in Table 2. The arithmetic mean value of cadmium in soil was 0.20 mg/kg, ranging from 0.084–4.52 mg/kg, which is larger than the Hebei soil background value (0.094 mg/kg) and the soil background value in China (0.097 mg/kg) [42]. In addition, two outliers were greater than the risk value of soil pollution in China’s agricultural land [48] (Chinese standard 0.6 mg/kg under pH > 7.5).

**Figure 2:**
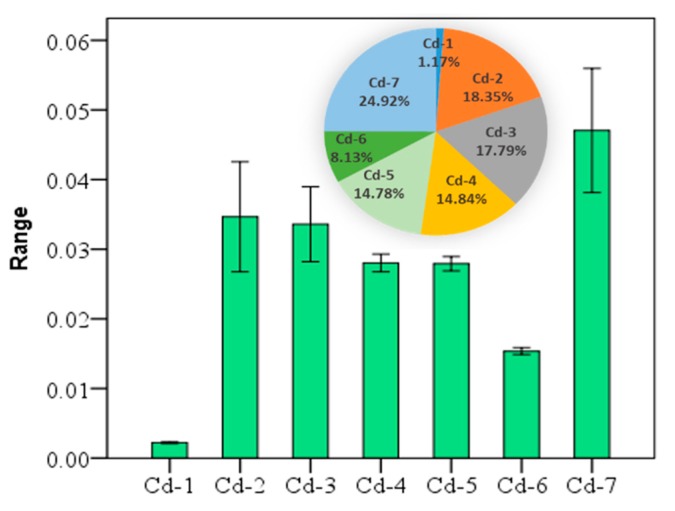
The mean ± standard error of seven fractions of cadmium (Cd) at the 95% confidence interval, and the percentage of each fraction of the total (unit: mg/kg).

The arithmetic mean concentration of the seven fractions of cadmium were 0.002 (Cd-1, water-soluble), 0.035 (Cd-2, exchangeable), 0.034 (Cd-3, carbonate-bound), 0.028 (Cd-4, humic acid-bound), 0.0279 (Cd-5, Fe-Mn oxide-bound), 0.015 (Cd-6, organic matter-bound), and 0.047 (Cd-7, residual), respectively. The seven fractions of Cd in soil are in the following descending order: Cd-7 > Cd-2 > Cd-3 > Cd-4 > Cd-5 > Cd-6 > Cd-1. According to the Cd values, the percentages of other fractions cannot be ignored either, except for Cd-1 and Cd-6 (Figure 2), where Cd may cause certain environmental risks.

**Figure 3:**
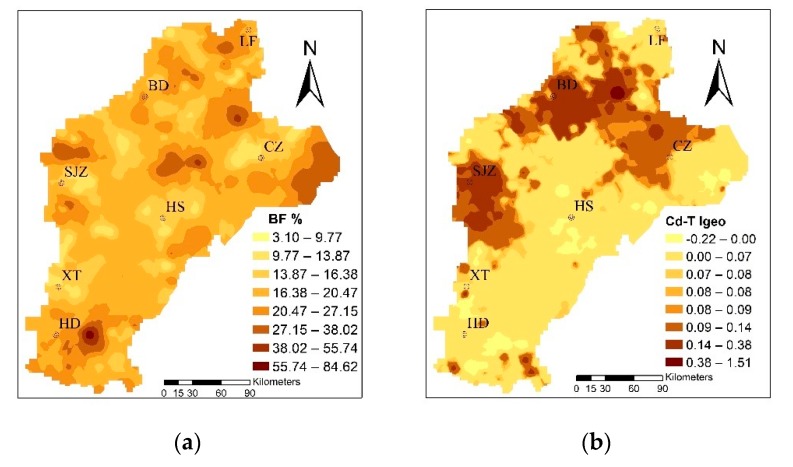
The distribution of the bioconcentration factor (BF%) of trace metals and the geoaccumulation index (I_geo_) of total Cd in soil (Cd-T).

The mean I_geo_ was 0.076 and, based on the standard classification, it showed unpolluted levels. However, the values ranged from −0.22 to 1.51, with 73.91% samples at the unpolluted to moderately polluted levels; thus, it could be seen that a human factor exists. According to the spatial distribution of Cd in soil (Figure 3), using the I_geo_ to evaluate the degree of pollution in the study area shows that the mild to moderate cumulative polluted areas were mainly concentrated in BD, SJZ, and the western CZ region. Domestic scholars Guo (2011), Zhang (2007), and Cui (2014) have hypothesized that the Cd in the study area is mainly derived from sewage irrigation, fertilizer, and long droughts in recent years. The study areas of agricultural water used for agricultural production, mainly from the groundwater and relatively close to the town region, gradually began to adopt sewage irrigation. Pollution of irrigation farmland, which was untreated for a long period of time, led to the Cd ion concentration in the farmland. In addition, in order to improve the production in rural areas, unreasonable use of fertilizers in farmland caused further Cd pollution [49,50,51]. Xiao et al. (2019) and Rao et al. (2018) showed that the long-term application of fertilizers, especially inorganic fertilizers, led to the enrichment of Cd in the cultivated soil of wheat, thus leading to the accumulation of Cd in wheat [8,17].

### 3.2. Cadmium Content in Wheat

The cadmium content ranged from 0.012–0.141 mg·kg^−1^, with an average value of 0.032 mg/kg and, when compared to the standard food limit (0.1 mg/kg) [52], three standard samples exceeding the acceptable limit of Cd content in wheat. The three sites corresponding to the content of the soil were 4.52, 0.22, and 0.13. Applying the Pearson correlation analysis (Table S2), the Cd content in wheat had a significant correlation with the total for Cd in soil, which was 0.307 at the *P* < 0.01 level. The speciation of cadmium with that in wheat had a significant correlation; the Cd-2, Cd-4, and Cd-7 fractions at the *P* < 0.01 level were 0.320, 0.300, and 0.390, respectively. From the wheat and three fractions of patterns that can be similarly distributed, we suggest that Cd-2, Cd-4, and Cd-7 migrated to wheat as the main source, and the change of environment was the most active in the environment, and the percentage of the three types of fractions was 58.12%, which easily migrates into, and can be absorbed and used directly by the plant.

The mean value of BF was 0.197, which is larger than the average value of the 10-year experimental field studied by Yang [53]. The enrichment capacity of wheat was strong; the range was from 0.0031 to 0.85, and the maximum was 274 times the minimum, showing that the enrichment capacity variety changed from soil to wheat. The BF was concentrated to the east of HD and LF, and to the east of CZ, as shown in Figure 3. A possible explanation for this is that the Cd-T is often only characterized in the soil, so that the presence of plants on the absorption of Cd enrichment depends on speciation rather than Cd-T [54,55]. Furthermore, the degree of contamination caused by Cd-T, which causes harm to crop growth and soil properties, mainly depends on the various fractions [56,57,58]; that is, different fractions determine the mobility and bioavailability of Cd, leading to different enrichment capacities.

### 3.3. Relation Between Cadmium in the Wheat–Soil System

**Figure 4:**
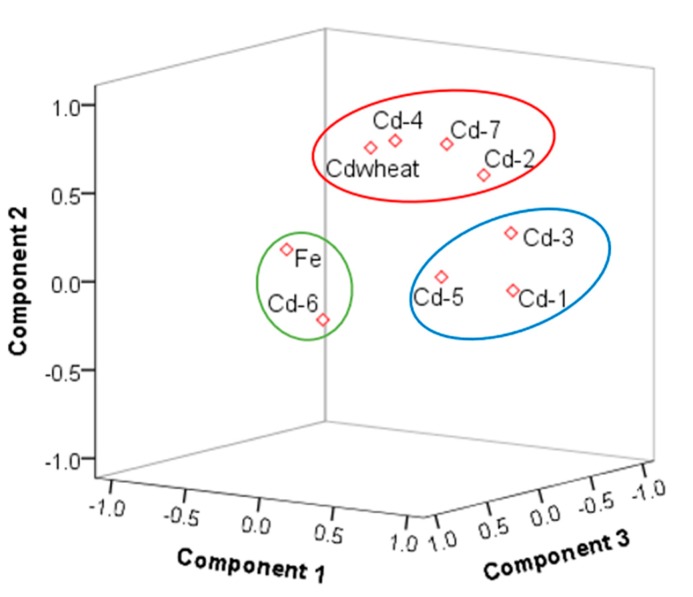
The three components plotted in rotated space (the circles represent the different source components).

**Figure 5:**
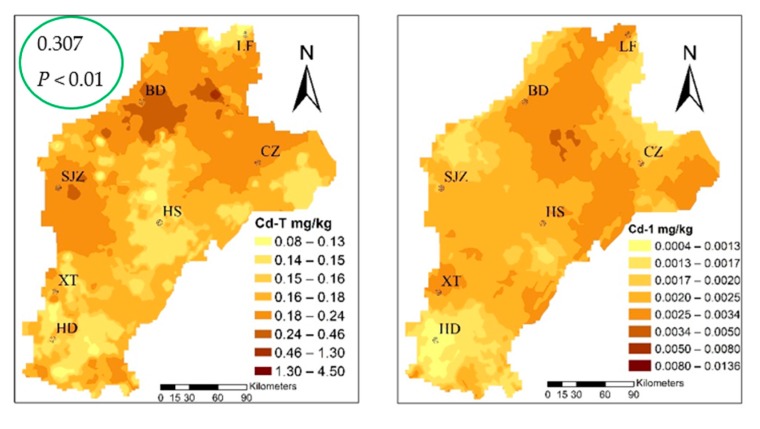

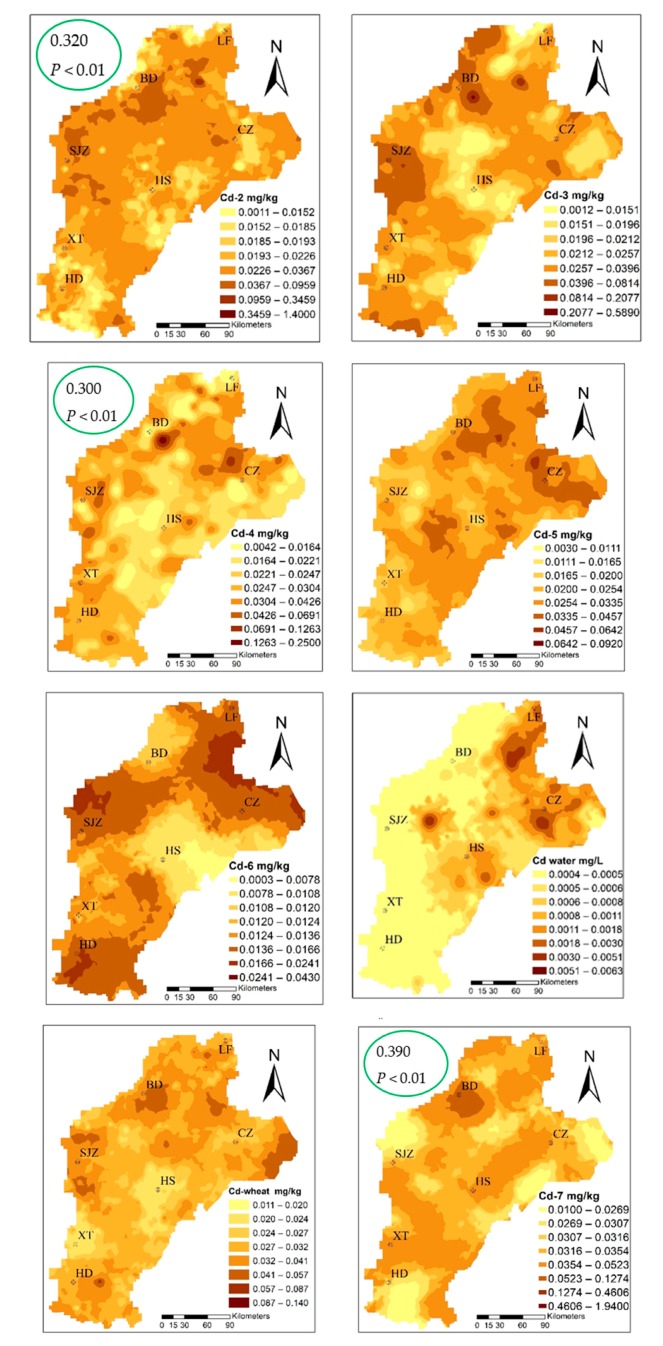
Spatial distribution of cadmium speciation and in multiple media (the green circles highlight the correlation coefficient between Cd-2, Cd-4, Cd-7, and Cdwheat).

The sources of cadmium were analyzed by PCA. The Kaiser–Meyer–Olkin (KMO) test of sampling adequacy was 0.7, Sig0.000; thus, the results of the PCA were reliable. The cumulative contribution rate of the PCA was approximately 70% (Tables S3 and S4). The mean of Fe was 15.55% (the mean of Fe_2_O_3_ was 4.49 mg/kg, standard deviation 0.698, consistent with normal distribution), and the coefficient of variation was low, which was defined as a natural source. We considered Fe_2_O_3_, Cd-T, and seven fractions in the PCA. The results of the rotated component matrix of Cd-T, Fe_2_O_3_ with Cd-6 came from natural sources. The concentration and distribution of Cd-6 in soil were determined by the soil-forming process, where Cd-1, Cd-3, Cd-5 belonged to a group and were formed by physical and chemical changes. Cd-2, Cd-4, Cd-7, and Cdwheat were points in the same group (Figure 4), showing that they belonged to the same origin, and further showing the migration and accumulation of Cd-2, Cd-4, and Cd-7 to wheat grain by physical and chemical processes. Moreover, the spatial distribution map had a similar pattern (Figure 5). In addition, the results combined with the SMLR analysis were as follows: Cd-2 = −0.018 + 0.307 Cd − 0.01 TOC., R^2^ = 0.99, *P* < 000; Cd-4 = 0.005 + 0.033 Cd − 0.011 TOC + 0.0001 clay, R^2^ = 0.53, *P* < 000; Cd-7 = −0.019 + 0.323 Cd, R^2^ = 0.86, *P* < 000. Cd-2, Cd-4, and Cd-7 were controlled by Cd-T, Clay, CeC, and TOC. These results are similar to Deng (2019), Shi (2019), and Zhu (2019) [22,59,60].

These study results are different from the research findings of other scholars [56,61,62]. Other scholars believe that the residue fraction belongs to the stable and difficult moving Cd, and the water-soluble fraction belongs to the active Cd and is easily absorbed by crops. However, the findings of this study showed that the residues migrated into the wheat grain, and the water-soluble fraction did not accumulate in the wheat grain. A better explanation is that the water soluble and residues of each trace metal have different migration characteristics under the influences of local farming conditions and physical and chemical factors. Factors such as a high temperature condition can lead to the release of potentially toxic trace elements, which become mobile in agricultural soils and then transfer to basic food crops [32].

In the future, we plan to conduct detailed analyses and research on the transformation and mobility mechanisms and physiological mechanisms of trace metal fractions, and simulate the change rule under different physicochemical environment conditions to find the best method to inhibit the migration and transformation of the trace metal fraction into wheat.

### 3.4. Cadmium Content in Atmospheric Deposition 

**Figure 6:**
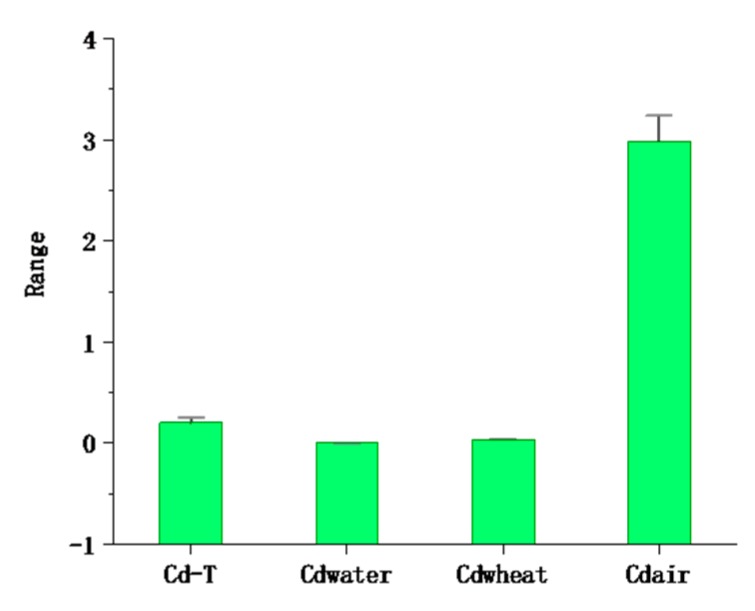
The mean ± standard error of cadmium in multiple media at the 95% confidence interval (the unit of Cd-T, Cdwheat, and Cdair is mg/kg, and the unit of Cdwater is mg/L).

The average content of cadmium in the atmospheric deposition in the study area was 2.99 mg/kg^−1^ and ranged from 0.47 to 7.87 mg/kg^−1^ (Table 2 and Figure 6). The average value was 31.8 times the local soil background value, and the samples exceeding the soil background value accounted for 99.13% of the total number of samples, far higher than GB 15618-2018 (pH > 7.5, 0.6 mg/kg) [48]; 15 times the mean (0.2 mg/kg) in the study area. The I_geo_ ranged from (mean value 1.21) 0.52 to 1.75, which indicates that there is a moderate pollution level of Cd in the atmosphere. This indicates that the Cd in the atmosphere is not caused by local soil but comes from manmade pollution. The input fluxes of Cd were 5.68 g/hm^2^ · a by the equation, far greater than the Beijing Plain (2.36), Changchun City (2.5), and Heilongjiang Province (1.46 g/hm^2^ · a) [63,64,65]; in addition, a large amount of Cd is imported into the soil from atmospheric deposition. Xia et al. (2014) found that the input fluxes of Cd deposited in the atmosphere in different regions were significantly different (Yi et al. (2018), Shi et al. (2019). It is believed that atmospheric deposition is the main origin of Cd accumulation in soil, rather than the application of phosphate fertilizers, which is related to the differences in farming systems and industrial level [66,67,68].

### 3.5. Cadmium Content in Underground Water

The primary drinking water sources and irrigation water in the research region were all from groundwater. The elemental Cd ranged from 0.0004 to 0.0063 mg/L, and the average was 0.0008 mg/L. According to the standard for groundwater quality (GB/T 14848-2017) [69], which is Cd ≤ 0.005 mg/L for groundwater used in centralized drinking water and industrial and agricultural water, 97.9% of the samples met the requirements, but 2.1% of the samples were higher than the drinking water standard. The WHO guideline is 0.003 mg/L, and drinking water is generally less than 0.001 mg/L [70,71]. The mean value of drinking water is at a safe level. However, 39 samples exceeded 0.001 mg/L, accounting for 16.39% of the total samples. Thus, there is some human influence.

### 3.6. Health Risks of Cadmium in Multiple Media

**Figure 7:**
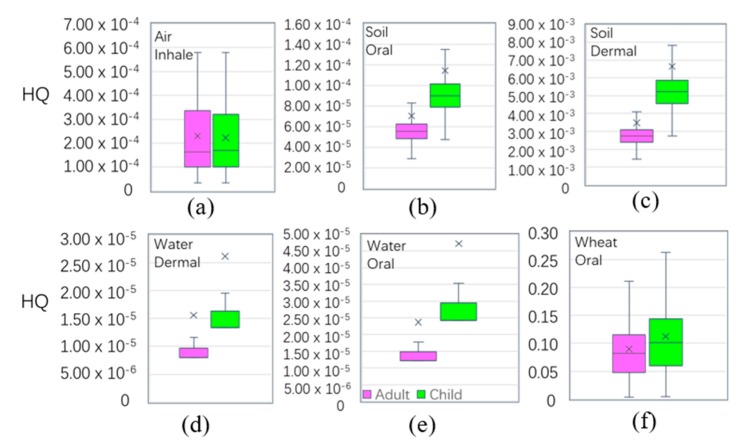
Boxplot of non-carcinogenic risk (hazard quotient) among the different exposure pathways (hazard quotient (HQ) is unitless).

In each medium (Table 3), the oral intake of ADD-a(adult’s ADD) and ADD-c(children’s ADD) in wheat was greater than those in other pathways, followed by the oral intake of water. The six pathways in the four media (wheat, soil, water, and air) in order were: wheat-oral > water-oral > soil-oral > air-inhale > soil-dermal > water-dermal. Figure 7 shows that the exposure risk via oral was more harmful than by other pathways, and that children > adults. Table 2 shows the average values of HQ in the main pathways of wheat, soil, water, and air. The mean values of the HQ of adults(HQ-a) and children (HQ-c) were 9.33 × 10^−2^ and 1.19 × 10^−1^, respectively, smaller than the national safe limited value of 1, respectively. The HQ values were higher in children than in adults through the six main exposure pathways in the four media. The HQ values were presented through the main exposure pathways of the four media through the six pathways, and the results were as follows: wheat-oral > soil-dermal > air-inhalation > soil-oral > water-oral > water-dermal. This suggests that wheat consumption through the oral ingestion pathway was the main pathway for Cd exposure. Therefore, the consumption of wheat by residents in the study area was the main cause of non-cancer risk. The results of wheat ingested were similar with domestic Kunshan (HQ-a 0.13, HQ-c 0.14) [72], higher than the overseas Semnan Province, Iran (HQ-a 0.05, HQ-c 0.03) [73], and Bangladesh (HQ-a 0.005) [74], leading to the main differences being due to the mean concentration of Cdwheat in the study area (0.032 mg/kg), which was higher than Iran (0.018 mg/kg), and Bangladesh (0.011 mg/kg). However, the HQ value of Cd in the four media were all less than the safety limit of ‘1’, indicating that Cd in the four media did not have a significant to non-carcinogenic risk to the human body. Compared with other places in terms of the HQ level of adults in agricultural soil, the results were smaller than Germany, 0.02 [75]; Canada, 0.001 [76]; Mexico, 0.034 [77], and South Africa, 0.036 [78].

**Figure 8:**
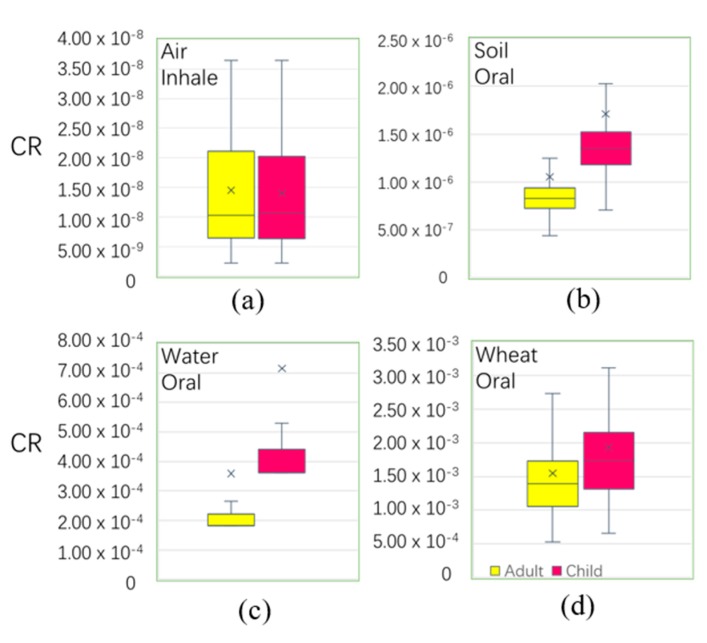
Boxplot of carcinogenic risk among different exposure pathways. (the total cancer risk (CR) is unitless).

According to the calculation results, the pathways of air and soil had not obviously affected the cancer risk to local residents. However, wheat by ingestion and underground water by ingestion were greater than the other pathways (Figure 8). The mean values of the total CR of adults and children were 1.91 × 10^−3^ and 2.65 × 10^−3^, which exceeded the tolerant limited range (10^−6^ < CR < 10^−4^) and could lead to cancer risk. Compound risk levels were caused by wheat when ingested (1.93 × 10^−3^ and 1.55 × 10^−3^ for children and adults, respectively) and by groundwater when ingested (7.15 × 10^−4^ and 3.59 × 10^−4^ for children and adults, respectively). In the predicted distribution of wheat and water by ingestion (Figure S1), the high value patterns of water CR were concentrated in LF, CZ, east of SJZ, and HS, and the high value patterns of wheat CR were concentrated in LF, BD, SJZ, HD, and east of CZ, where residents paid more attention to water and wheat when ingestion is a high risk to health. 

In addition, according to the FAO/WHO, the recommended tolerable daily Cd uptake is 10–35 μg/d [26]. If only water and wheat are taken into account, the range of daily Cd absorption in adults can be calculated from 3.5 to 43.5 μg/d, and it can be seen that the results of part of the Cd also exceeded the absorption tolerant limit.

#### Health Risk Uncertainty Analysis

In this research, the total concentrations of selected Cd were analyzed from underground water, soil, wheat, and atmospheric deposition, collected from the survey region to calculate the overall health risks to the local population. Trace metals not only exist in a single medium, but also exist in wheat. Therefore, those trace metals will lead to compound health risks of pollutants when ingested. In addition, the degree of risk is closely related to consumer habits, lifestyles, occupational types, and other factors [22,24,26,47], which requires a more complex exposure assessment method to calculate the daily exposure to pollutants. All these factors require further study. Previous studies have shown that contaminated crops from Cd once ingested could significantly reduce the average human life by 9 to 10 years [79]. However, the results for Cd are valuable for residents to assess the Cd risk from multiple media to human health. 

## 4. Conclusions

In the studied area, the concentration of seven types of fractions of bulk soil were determined, and the order was Cd-7, residual > Cd-2, exchangeable > Cd-3, carbonate-bound > Cd-4, humic acid-bound > Cd-5, Fe-Mn oxide-bound > Cd-6, organic matter-bound > Cd-1, water-soluble. Through correlation and PCA, we determined that the Cd in wheat mainly came from Cd-2, Cd-4, and Cd-7 (accounting for 58.11% of the total) migration. The results imply that Cd poses an ecological risk for local agricultural soil. The relationship was established by SMLR and indicates that the Cd content of Cd-2, Cd-4, and Cd-7 is mainly determined by clay, CeC, and TOC. Based on these results, we plan to deeply research the formation and mobility mechanism of each fraction in the future. The mean Cd concentration was in the order air > soil > wheat > water (Figure 6), and the input flux of Cd was 5.68 g/hm^2^ · a, indicating that Cd in atmospheric deposition is a primary contributor to its accumulation in soil. The results of the total human health risk in multiple media showed that although the concentration of atmospheric deposition was very high, it was not a significant risk to human health. More attention should be paid to the harm caused by orally ingesting wheat and water to human health. Local residents are advised to improve the structure of staple foods and eat more corn flour with a low trace metals content as the staple food. The results provide valuable information for the local treatment of Cd pollution in multiple media.

## Figures and Tables

**Table 1:** Calculation formula of daily intake via various exposure pathways in multiple media.

Media	Exposure pathway	Calculation formula	Number
Soil	Ingestion by Oral	$ADD_{ingestion}=\frac{CS\times IRS\times EF\times ED}{BW\times AT}\times 10^{-6}$	(8)
	Dermal	$ADD_{dermal}=\frac{CS\times SA\times AF\times ABS\times EF\times ED}{BW\times AT}\times 10^{-6}$	(9)
Wheat	Ingestion by Oral	$ADD_{ingestion}=\frac{Cwheat\times IRwheat\times EF\times ED}{BW\times AT}$	(10)
Underground water	Ingestion by Oral	$ADD_{ingestion}=\frac{Cwater\times IRwater\times EF\times ED}{BW\times AT}$	(11)
	Dermal	$ADD_{dermal}=\frac{Cwater\times SA\times AF\times ABS\times EF\times ED}{BW\times AT}\times 10^{-6}$	(12)
Atmosphere deposition	Inhale	$ADD_{inhale}=\frac{Ca\times ET\times EF\times ED}{PEF\times 24\times AT}$	(13)

**Table 2:** Concentration statistics for each medium and seven types of fractions of Cd elements (unit: mg/kg, except for water: mg/L).

items	Minimum	Maximum	Mean	Std. Deviation	Coefficient of Variation	Skewness	Kurtosis
Cd-1	4.00 × 10^−4^	2.50 × 10^−2^	2.20 × 10^−3^	2.00 × 10^−3^	8.00 × 10^−1^	9.57	120
Cd-2	1.10 × 10^−3^	1.40	3.47 × 10^−2^	1.20 × 10^−1^	3.47	10.5	112
Cd-3	1.20 × 10^−3^	1.15	3.36 × 10^−2^	8.20 × 10^−2^	2.44	11.9	156
Cd-4	4.20 × 10^−3^	2.50 × 10^−1^	2.80 × 10^−2^	1.90 × 10^−2^	6.90 × 10^−1^	6.86	73.8
Cd-5	3.00 × 10^−3^	1.30 × 10^−1^	2.79 × 10^−2^	1.60 × 10^−2^	5.60 × 10^−1^	2.19	8.80
Cd-6	3.00 × 10^−4^	4.30 × 10^−2^	1.54 × 10^−2^	7.00 × 10^−3^	4.90 × 10^−1^	1.10	1.35
Cd-7	1.00 × 10^−2^	1.94	4.71 × 10^−2^	1.36 × 10^−1^	2.88	12.7	171
Cd-T	8.40 × 10^−2^	4.52	2.03 × 10^−1^	3.90 × 10^−1^	1.92	10.6	111
Cdwheat	1.20 × 10^−2^	1.40 × 10^−1^	3.22 × 10^−2^	1.60 × 10^−2^	5.10 × 10^−1^	2.87	12.4
Cdwater	4.00 × 10^−4^	6.30 × 10^−3^	8.00 × 10^−4^	1.00 × 10^−3^	1.30	3.44	12.5
Cdair	4.70 × 10^−1^	1.23 × 10^2^	5.52	17.4	3.16	6.79	46.7

**Table 3:** Exposure to different media, non-carcinogenic risk, and carcinogenic risk (-a for adult, -c for children).

Media	Pathways	ADD-a	ADD-c	HQ-a	HQ-c	CR-a	CR-c
Air	Inhale	2.30 × 10^−9^	2.22 × 10^−9^	2.30 × 10^−4^	2.22 × 10^−4^	1.45 × 10^−8^	1.4 × 10^−8^
Soil	dermal	3.47 × 10^−8^	6.65 × 10^−8^	2.91 × 10^−3^	6.64 × 10^−3^	_	_
	Ingestion by oral	7.05 × 10^−8^	1.14 × 10^−7^	7.05 × 10^−5^	1.14 × 10^−4^	1.06 × 10^−6^	1.72 × 10^−6^
Water	Ingestion by oral	2.37 × 10^−5^	4.72 × 10^−5^	2.37 × 10^−5^	4.72 × 10^−5^	3.59 × 10^−4^	7.15 × 10^−4^
	dermal	1.56 × 10^−10^	2.62 × 10^−10^	1.56 × 10^−5^	2.62 × 10^−5^	_	_
Wheat	Ingestion by oral	1.03 × 10^−4^	1.29 × 10^−4^	9.00 × 10^−2^	1.10 × 10^−1^	1.55 × 10^−3^	1.93 × 10^−3^
total		**Total of ADD-a**	**Total of ADD-c**	**HI**		**TCR**	
		1.27 × 10^−4^	1.76 × 10^−4^	9.33 × 10^−2^	1.19 × 10^−1^	1.91 × 10^−3^	2.65 × 10^−3^

## Supplementary Materials

The following are available online at https://www.mdpi.com/1660-4601/16/13/2269/s1, Table S1: The values and significance of the parameters for the health risk calculations. Table S2: The correlation coefficients between Cd-T and the seven fractions and Cdwheat. Table S3: Total variance explained by the PCA. Table S4: Rotated component matrix a of 1,2,3 components. Figure S1: The spatial distribution of cancer risk to adults and children predicted by water and wheat when ingested.
