# Supplementary material for: Concentration, Source, and Total Health Risks of Cadmium in Multiple Media in Densely Populated Areas, China
Source: Int J Environ Res Public Health. 2019 Jun 27;16(13):2269. doi: 10.3390/ijerph16132269 (PMC6651708; doi:10.3390/ijerph16132269)
Supplement: Supplementary file 1 [file ijerph-16-02269-s001.pdf]

Supplementary Materials

Table S1. The values and significance of parameters of health risk calculating.

parameters	Unit and value	significance	reference
Cwater	mg/L	Concentration in groundwater	[45]
CS	mg/kg	Exposure-point concentration	[45]
EF	365 d/a	Exposure frequency	[45]
ED	adults 70 a; children 6a	Exposure duration	[45]
ET	24 h/d	Exposure frequency	[45]
AT	365 × ED day	Averaging time for (non)carcinogens	[45]
BW	adults: 70 kg and children: 18 kg	Bodyweight	[46]
SA	adults 5700 $cm^2 \cdot day^{-1}$, children and 2800 $cm^2 \cdot day^{-1}$	Exposed skin area	[45]
AF	adults 0.07 $mg \cdot cm^{-2}$, children 0.02 $mg \cdot cm^{-2}$	Adherence factor	[45]
ABS	0.001	Dermal absorption fraction	[45]
PEF	1.36×10^9 $m^3 \cdot kg^{-1}$	Particle emission factor	[6]
CF	10^{-6} $kg \cdot mg^{-1}$	Units conversion factor	[45]
IRS	adults 100 $mg \cdot d^{-1}$, children and 200 $mg \cdot d^{-1}$	Ingestion rate	[45]
IRwheat	adults 0.225 $kg \cdot d^{-1}$, children 0.075 $kg \cdot d^{-1}$	Ingestion rate of wheat	[34]
IRwater	adults 1.82 $L \cdot person^{-1} \cdot d^{-1}$, children 1.06 $L \cdot person^{-1} \cdot d^{-1}$	Ingestion rate of water	[47]
RFD	RFD ingestion: 0.001 $mg \cdot kg^{-1} \cdot d^{-1}$, RFD inhale: 0.00001 $mg \cdot kg^{-1} \cdot d^{-1}$, RFDdermal: 0.00001 $mg \cdot kg^{-1} \cdot d^{-1}$	Chronic reference	[45]
SF	SFingestion: 15$kg \cdot d \cdot mg^{-1}$, SFinhale: 6.3 $kg \cdot d \cdot mg^{-1}$.	Slope factor	[45]

Table S2. The correlations coefficient between Cd-T and 7 fractions and Cdwheat.

	Cdwheat	Cd-T	Cd-1	Cd-2	Cd-3	Cd-4	Cd-5	Cd-6	Cd-7
Cdwheat	1								
Cd-T	0.307**								
Cd-1	0.044	0.625**	1						
Cd-2	0.320**	0.995**	0.604**	1					
Cd-3	0.141*	0.894**	0.780**	0.874**	1				
Cd-4	0.300**	0.686**	0.182**	0.669**	0.439**	1			
Cd-5	0.121	0.427**	0.416**	0.402**	0.391**	0.240**	1		
Cd-6	0.013	0.056	0.018	0.049	0.042	0.009	0.051	1	
Cd-7	0.390**	0.927**	0.379**	0.932**	0.675**	0.756**	0.317**	0.023	1

**. Correlation is significant at $P < 0.01$ *. Correlation is significant at $P < 0.05$ level. Cd-1 water-soluble, Cd -2 exchangeable, Cd -3 carbonate-bound, Cd -4 humic acid-bound, Cd -5 Fe-Mn oxide-bound, Cd -6 organic matter-bound, Cd -7 residual, Cd-T total content

Table S3. Total Variance Explained of PCA.

Component	Initial Eigenvalues			Extraction Sums of Squared Loadings			Rotation Sums of Squared Loadings		
	Total	% of Variance	Cumulative %	Total	% of Variance	Cumulative %	Total	% of Variance	Cumulative %
1	3.923	43.594	43.594	3.923	43.594	43.594	2.855	31.717	31.717
2	1.286	14.294	57.888	1.286	14.294	57.888	2.318	25.756	57.474
3	1.054	11.708	69.596	1.054	11.708	69.596	1.091	12.123	69.596
4	0.953	10.592	80.188						
5	0.787	8.741	88.929						
6	0.609	6.770	95.698						
7	0.252	2.805	98.503						
8	0.123	1.367	99.870						
9	0.012	0.130	100.000						

Extraction Method: Principal Component Analysis.

Table S4. Rotated Component Matrix[a] of 1,2,3 components.

elements	Component 1	Component 2	3Component 3
Cd1	0.894	-0.016	-0.060
Cd2	0.740	0.630	0.002
Cd3	0.886	0.309	-0.052
Cd4	0.260	0.801	0.172
Cd5	0.601	0.069	0.212
Cd6	0.128	-0.152	0.683
Cd7	0.515	0.788	0.038
Cdwheat	-0.073	0.693	-0.067
Fe2O3	-0.082	0.231	0.733

Extraction Method: Principal Component Analysis.

Rotation Method: Varimax with Kaiser Normalization.

[a]. Rotation converged in 5 iterations.

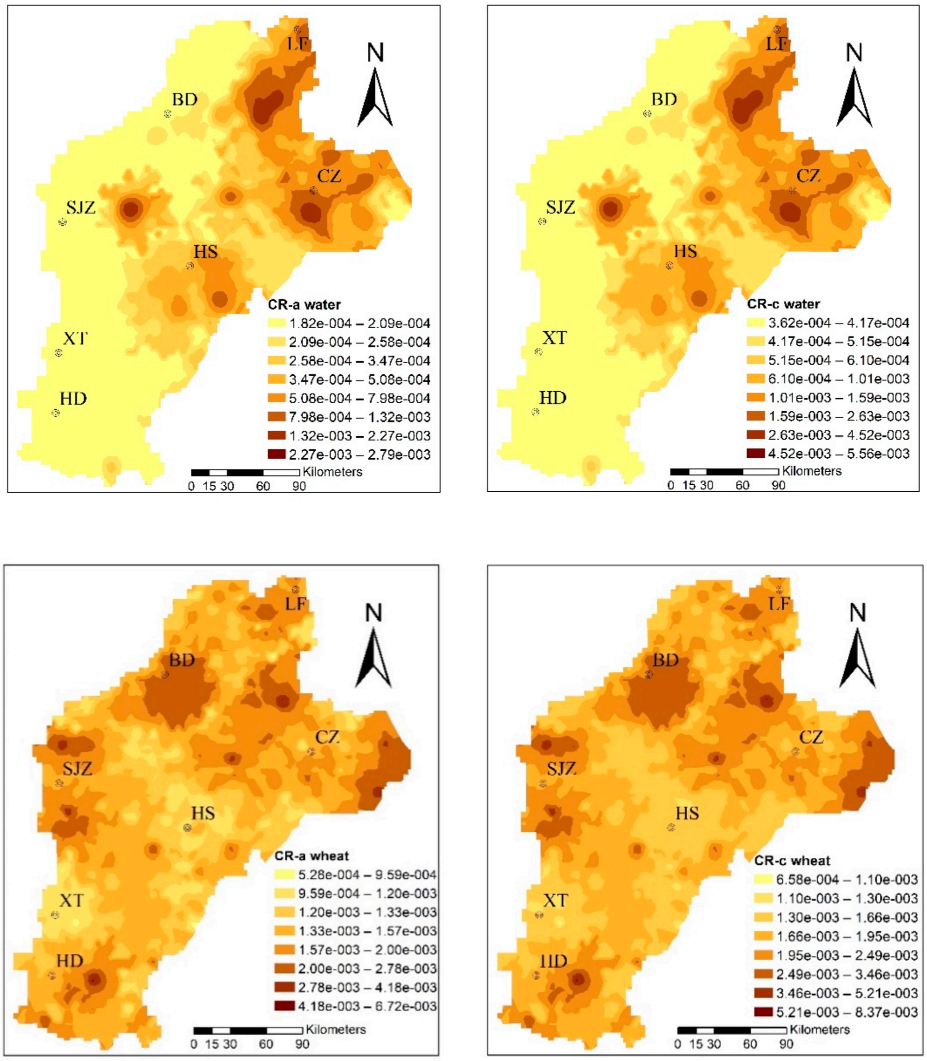

FigureS1. The spatial Distribution of cancer risk to adults and children predicted by water and wheat when ingestion.
